# Comparison of Non-Surgical Methods for Implant Surface Treatment in Simulated Bone Resorption Patterns: An In Vitro Study

**DOI:** 10.3390/jcm14207244

**Published:** 2025-10-14

**Authors:** Luca Sbricoli, Gaia Petrini, Alvise Camurri Piloni, Edoardo Stellini, Eriberto Bressan, Riccardo Favero

**Affiliations:** Department of Neurosciences, School of Dentistry, University of Padua, Via Giustiniani 2, 35100 Padua, Italy; gaia.petrini@outlook.com (G.P.); alvise.camurripiloni@gmail.com (A.C.P.); edoardo.stellini@unipd.it (E.S.); eriberto.bressan@unipd.it (E.B.); rickyfavero@msn.com (R.F.)

**Keywords:** dental implants, dental hygiene, peri-implantitis, implant surface decontamination

## Abstract

**Background**: Peri-implantitis is the leading cause of implant failure, with a reported prevalence of 22–45%. Effective removal of bacterial biofilm from the implant surface is critical to non-surgical therapy. This study aimed to assess the efficacy of different implant surface cleaning methods across various bone defect configurations, considering operator experience. **Methods**: Thirty-six dental implants were coated to simulate biofilm, mounted in resin blocks with bone defects of varying geometries, and covered with silicone to simulate soft tissue. Three operators with differing levels of experience treated the implant surfaces using four instruments: a titanium curette (TiCu), ultrasonic scaler (US), titanium brush (TiBr), and air abrasion with erythritol (AirPo). Each combination was tested in triplicate. Implants were photographed and analyzed with dedicated software to quantify cleaning efficacy. **Results**: The expert dentist achieved the highest average cleaning efficacy (36.6%). The most effective tools were the titanium brush (37.2%) and ultrasonic scaler (35.0%), followed by the titanium curette (28.1%) and air-abrasion (22.9%). The first two instruments were the least operator-dependent. Among the defect types, the 60° defect was the easiest to clean. Complete implant surface decontamination was not achieved in any scenario. **Conclusions**: Ultrasonic scalers and titanium brushes demonstrated the highest and most consistent cleaning efficacy, independent of operator skill level. Sixty-degree defects were the most amenable to cleaning. These findings underscore the need to tailor decontamination approaches based on defect geometry and to consider combining non-surgical methods with adjunctive or surgical interventions, which may ultimately enhance clinical decision-making and improve treatment outcomes.

## 1. Introduction

Implant therapy has very high success rates for restoring edentulous areas. With the number of dental implants increasing every year, the incidence of biological complications, represented by mucositis and peri-implantitis, has also increased. While mucositis appears to be a reversible inflammatory pathology, peri-implantitis represents a more complex clinical challenge. A recent literature review shows peri-implantitis to have an estimated prevalence of 22% to 45% at the patient level, and 11% to 13% at the implant level [1,2,3]. Although the etiopathogenesis of peri-implant disease is multifactorial and still unclear, the formation of a pathogenic biofilm on the implant surface plays a pivotal role in the development and progression of peri-implantitis [4,5,6].

Decontamination of the implant surface is, therefore, considered to be one of the cornerstones of peri-implantitis therapy [7,8]. Many instruments have been proposed for cleaning the implant surface: ultrasonic debridement [9], titanium brushes [10,11], titanium or carbon fiber curettes [12,13], and air-polishing devices [14,15,16]. Recent in-vitro studies have reported highly varied success rates, ranging between 12 and 74% of the surface cleaned [17,18].

Another challenging aspect of non-surgical therapy for peri-implantitis is access to the surface to be treated. Given the diverse morphologies of bone defects, non-surgical therapy is currently not able to provide predictable and successful results, especially in more complex cases [19,20]. Peri-implant bone defects with a narrower conformation provide less space for larger instruments and offer less freedom of maneuver to the operator. To facilitate mechanical decontamination a surgical approach may be necessary. Recent studies on peri-implant treatments consistently indicate that even with optimal access and visibility, mechanical decontamination remains challenging and is highly dependent on the operator’s expertise [16].

The aim of the present work is to determine the most effective tool for cleaning the implant surface according to the conformation of the defect and the experience of the operator. The novelty of this study lies in the combined evaluation of defect morphology, instrument type, and operator experience, an approach not previously investigated in similar in vitro models.

## 2. Materials and Methods

### 2.1. Study Design

We carried out a single-center in vitro study using experimental resin models created to reproduce a real clinical situation. Specifically, 360° models of bone resorption around dental implants at angles of 30°, 60° and 90° were created. A hole was drilled in each model in which to insert the implant. Thirty-six dental implants (ITS ITALY Essemme Components Srl, Vigonza (PD), Italy), 4 mm in diameter and 13 mm in length, were used for the study. The number of implants (n = 36) was chosen to ensure feasibility and reproducibility in line with previously published in vitro models [16,17,18]. No formal power calculation was performed given the exploratory nature of the study.

**Figure 1:**
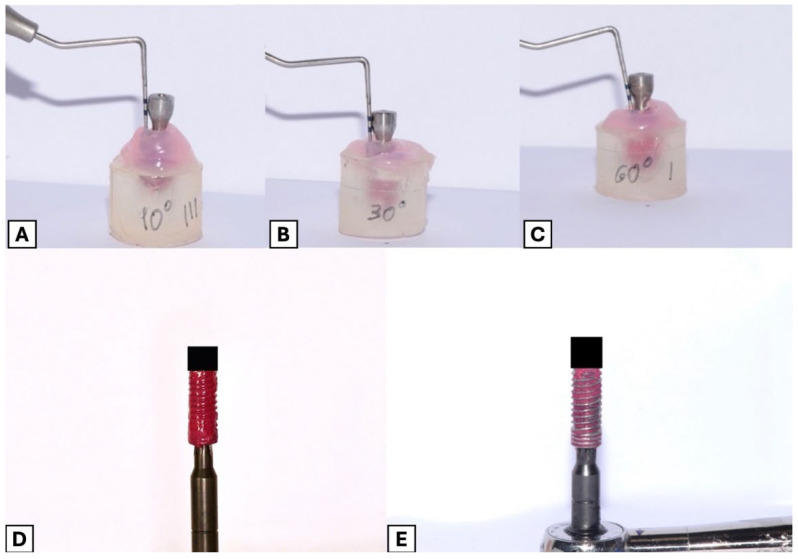
(**A**) study model with 90° defect simulation. (**B**) study model with 30° defect simulation. (**C**) study model with 60° defect simulation. (**D**) Initial photo of a fully colored implant. (**E**) Photo of implant after mechanical treatment with an ultrasonic scaler.

Each implant was painted with fuchsia nail polish to recreate the biofilm, using the method described by Tuchscheerer et al. [16]. This approach allows standardized coating and reproducible quantification of surface cleaning, although it does not reproduce the full biological and structural complexity of a real biofilm, which must be acknowledged as a limitation. The soft tissue around the implants was reproduced with a pink silicone-based prosthesis liner (Ufi Gel, VOCO GmbH, Cuxhaven, Germany), maintaining the same probing depth in each sample (Figure 1A–C).

### 2.2. Procedures

Three operators were chosen: G.P., a dental hygiene student (STUD); A.S., a dental hygienist with more than 10 years’ experience (IGEXP); and L.S., a dentist with more than 10 years’ experience (ODEXP). The operators were instructed to clean the implant surface to the best of their ability, without prior knowledge of the defect configuration. Each operator cleaned 3 different defects on 3 different implant models with 4 different instruments: titanium curette (TiCu), ultrasonic scaler (US), titanium brush (TiBr) and air-polishing with erythritol (AirPo), for a total of 108 measurements. The operators were given three minutes to carry out each task, after each of which the simulated biofilm was reapplied. The inclusion of operators with different training levels was intended to provide an overview of how operator skill might influence cleaning efficacy. This design aimed to capture a realistic spectrum of clinical variability rather than to compare professional categories per se. The three-minute limit was chosen based on previously published experimental models with similar designs. Moreover, this timeframe could represent a reasonable compromise to ensure standardization of the procedures while still reflecting a realistic clinical condition.

### 2.3. Measurements

After each treatment each implant was carefully removed and photographed at 25 cm with a reflex camera fitted with a 105 mm macro lens. The images were cropped to 4:5 with the size of the implant as a reference, then analyzed with the Image Color Summarizer software v0.80 (Genome Sciences Center) to measure the total surface and the surface remaining colored. The following parameters were set on the program: output format: html; statistics: color clusters; number of color clusters: 8; delimiter: space; precision: vhigh (200 px) (Figure 1D,E). This automated approach eliminates subjective interpretation and was therefore considered sufficient without additional inter- or intra-rater reliability testing, in line with similar published models [16,17,18].

### 2.4. Statistical Analysis

The data obtained from the experimental phase were entered into a database (Microsoft Excel v. 16, Microsoft Corporation, Redmond, WA, USA), then analyzed after dividing them into groups according to the relationships to be investigated. The variables were defined on the basis of the descriptive statistics and reported as frequencies or percentages as appropriate. Normality of data distribution was verified with the Shapiro–Wilk test and homogeneity of variances with Levene’s test. The pooled data were subjected to a two-way ANOVA to ascertain whether there were any significant differences between the 3 different groups at a significance level of *p* < 0.05. When significant differences were detected, Bonferroni-adjusted post hoc tests were performed.

## 3. Results

We took 108 measurements, all of which (100%) were considered valid. Statistical analysis revealed significant differences between groups in relation to both operator experience and instrument efficacy.

**Figure 2:**
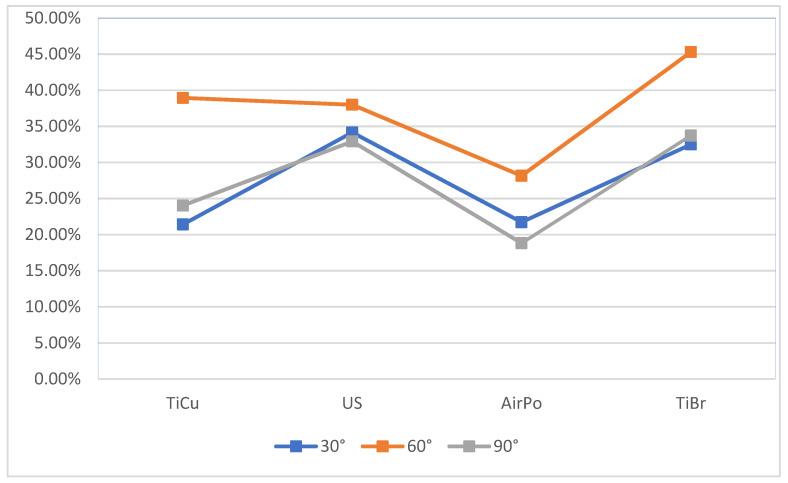
Percentage of the implant cleaned according to the level of bone defect, with the use of different instruments (titanium curette, ultrasonic scaler, air-polishing with erythritol, titanium brush).

Overall, ODEXP achieved an average cleaning efficacy of 36.6%, IGEXP 29.7%, and STUD 26.1%. Regarding the instruments, regardless of the operator’s experience and the type of defect, the ultrasonic scaler and the titanium brush were on average the most effective (35.01% and 37.16%, respectively), while the titanium curettes (28.14%) and erythritol air-polishing (22.89%) were less effective despite attaining high individual values that deviated greatly from the average during the experiment. Regarding the cleanability of the defects according to the resorption pattern, the 60° defect was found to be, on average, more cleanable than the 30° and 90° defects (Figure 2). Results are summarized in Table 1.

**Figure 3:**
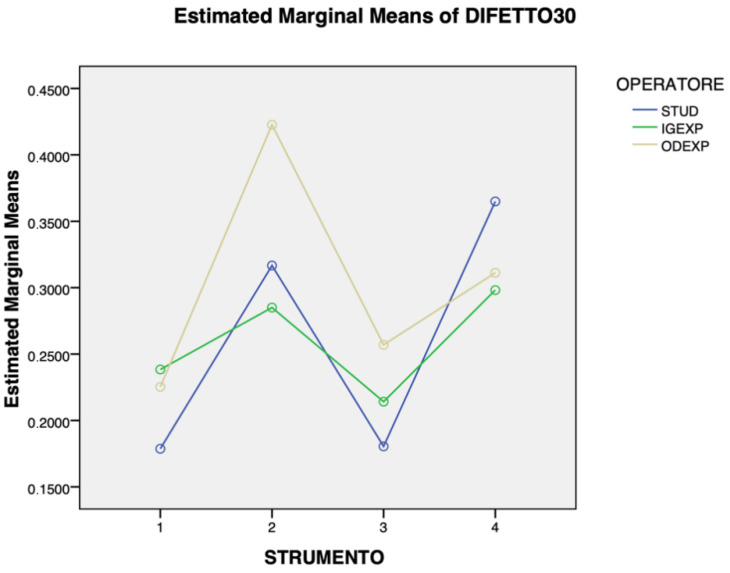
Relationship between the type of instrument used and the operator’s experience in treating 30° defects (STUD = dental hygiene student, IGEXP = expert hygienist (>10 years’ experience), ODEXP = expert dentist (>10 years’ experience), 1 = titanium curette, 2 = ultrasonic scaler, 3 = air-polishing with erythritol, 4 = titanium brush). TiBr reached 11% more cleaning than AirPo (*p* = 0.02).

**Figure 4:**
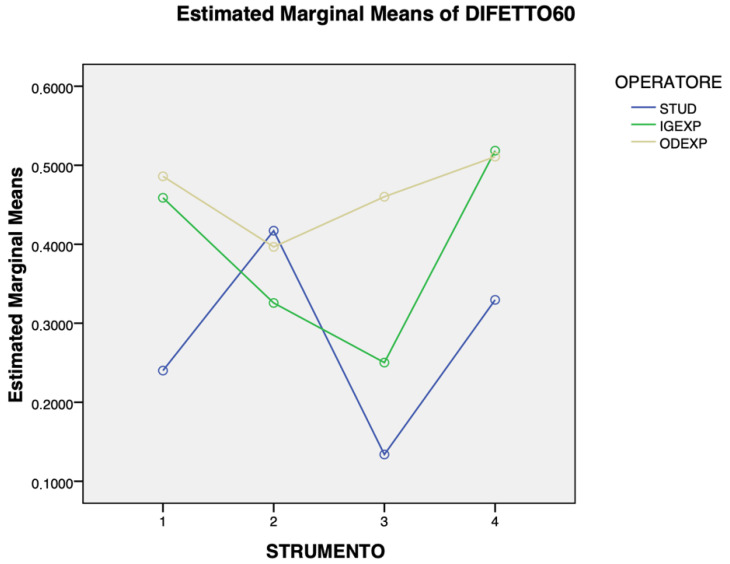
Relationship between the type of instrument used and the operator’s experience in treating 60° defects (STUD = dental hygiene student, IGEXP = expert hygienist (>10 years’ experience), ODEXP = expert dentist (>10 years’ experience), 1 = titanium curette, 2 = ultrasonic scale, 3 = air-polishing with erythritol, 4 = titanium brush). TiBr reached 17% more cleaning than AirPo (*p* = 0.03) and ODEXP performed 18% better than STUD (*p* = 0.01).

**Figure 5:**
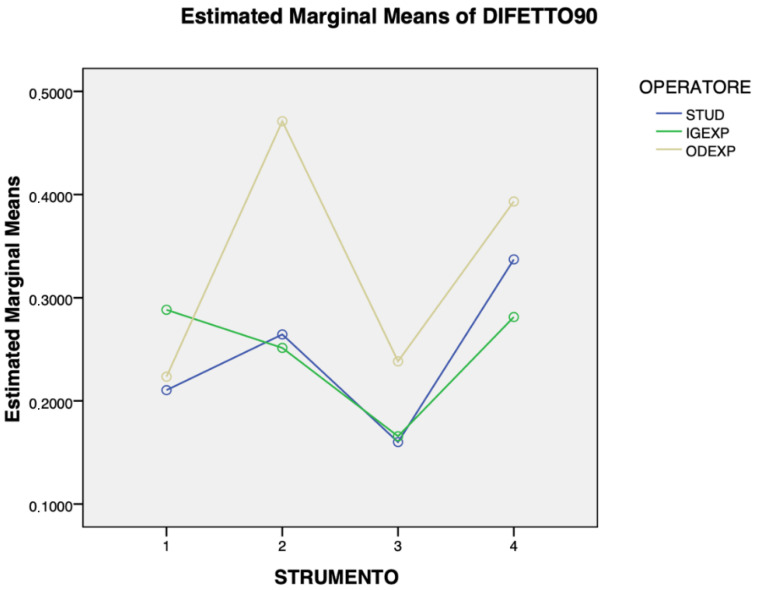
Relationship between the type of instrument used and the operator’s experience in treating 90° defects. (STUD = dental hygiene student, IGEXP = expert hygienist (>10 years’ experience), ODEXP = expert dentist (>10 years’ experience), 1 = titanium curette, 2 = ultrasonic scale, 3 = air-polishing with erythritol, 4 = titanium brush). US reached 9% more cleaning than TiCu (*p* < 0.01), TiBr 10% more cleaning than TiCu (*p* = 0.045), US 14% more cleaning than AirPo (*p* < 0.01) and TiBr performed 15% more cleaning than AirPo (*p* < 0.01).

Figure 3, Figure 4 and Figure 5 show the overall results regarding cleaning efficacy according to operator, instrument, and defect angle. For the 30° defect, the expert dentist generally outperformed the expert hygienist and the student, although the student achieved superior outcomes with the ultrasonic scaler and titanium brush compared to both more experienced operators. A statistically significant mean difference of 11% of cleaning was detected between TiBr and AirPo (*p* = 0.02). No other statistically significant differences were found in the 30° defect cleaning. With the 60° defect, the expert dentist obtained better and more consistent results than the other two operators, whose performance was highly variable, especially with AirPo, where their results differed greatly from the dentist’s. The only instrument where all three operators had similar results with this defect was US. A mean difference of 17% of cleaning between TiBr and AirPo (*p* = 0.03) and a mean difference of 18% between STUD and ODEXP (*p* = 0.01) were detected. No other statistically significant differences were found in the 60° defect cleaning. Finally, the results for three operators followed a similar trend with the 90° defects, with the ODEXP achieving the best results, and IGEXP and STUD performing similarly. Mean differences of 9% of cleaning between TiCu and US (*p* < 0.01), of 10% between TiCu and TiBr (*p* = 0.045), of 14% between US and AirPo (*p* < 0.01) and of 15% between AirPo and TiBr (*p* < 0.01) were detected. No other statistically significant differences were found in the 90° defect cleaning.

## 4. Discussion

The present study sought to determine the most effective method of cleaning the implant surface according to the type of bone defect and the operator’s experience. The aim of the study was to compare the effectiveness of four different implant cleaning methods at three levels of bone resorption (30°, 60°, and 90°) using an in vitro model. Unlike previous similar studies [9,19,20], we decided to include an additional variable, namely the different levels of experience and manual skills of the operator. By simultaneously addressing operator experience, defect configuration, and instrument type, our design provides a more comprehensive and clinically relevant assessment than prior studies. This approach is consistent with previous research showing that operator skill significantly affects debridement outcomes [21]. Our aim was to encompass this variability to highlight which instruments may perform more consistently regardless of operator experience. To this end, three different operators (a dentist with more than ten years’ experience, an expert hygienist with more than 10 years’ experience, and a dental hygiene student) carried out the tasks to ascertain whether one instrument performs better than the others, even when used by a less experienced operator, with the aim of improving cleaning efficacy. Regardless of the type of instrument used and the degree of bone defect, color residues were present in all the implants analyzed. In fact, overall, the percentage cleaned was never above 50%, which is in line with previous studies that report that, on average, between 39% and 89% of the implant surface was cleaned [22,23]. This finding is concerning, as it suggests that adequate decontamination of the implant surface may not be feasible without surgical access.

When we compared the efficacy of the different cleaning devices, we found that titanium brushes and ultrasonic scalers achieved statistically better results than erythritol air-polishing and titanium curettes. This result, when taken together with the results of the comparative analysis of the experiments carried out by the different operators, clearly shows that these two instruments perform better than the others, regardless of the operator’s experience and manual skills, and are therefore the simplest to use and the most effective.

The efficacy of ultrasonic scalers and titanium brushes in decontaminating the implant surface is also confirmed by Shwartz et al. [11]. Conversely, Persson et al. [22] and Karring et al. [23] reported comparable outcomes between titanium curettes and ultrasonic scalers.

In marked contrast to what emerges from the present study are the data reported by Iatrou et al. [20], who obtained better results with air-polishing (in this case with glycine instead of erythritol) than with curettes and ultrasonic scalers, and found that it required less manual skill. A systematic review by Louropoulou et al. [9] found that, although good results are obtained with ultrasonic scalers and titanium brushes, air abrasion is the most effective in cleaning the implant surface. In the present study, however, the erythritol air-flow was the instrument that performed the least well with all bone defects and in the hands of all the operators (except when used on 60° defects by the most experienced operator). These discrepancies may be explained by differences in the type of powder used (glycine or tricalcium phosphate vs. erythritol in our model), variations in air-polishing devices, and differences in experimental design (e.g., defect morphology, duration of application, operator protocols). Furthermore, while some studies employed flat discs or more accessible geometries, our setup reproduced three-dimensional peri-implant defects, which may have disadvantaged the air-polishing approach.

Differences emerged in the efficacy of the individual instruments, with the ultrasonic scaler and titanium brushes performing best, followed by the curettes and erythritol air-flow. We also found the former instruments to be the least operator-dependent and therefore the most suitable for use where the treatment is to be performed by a general operator whose level of experience is unknown or who is known to be inexperienced. Even where the treatment is performed by an expert operator, the instruments that achieve the highest cleaning percentages are the two aforementioned devices, although good results can also be obtained with titanium curettes in the hands of an expert, such as a professional hygienist. In fact, the best results with titanium curettes are obtained when they are used by an experienced hygienist, probably because these professionals routinely use these instruments in clinical practice and therefore with greater expertise. Although our in vitro model allowed standardization and control of key variables, the direct clinical translation of these results requires caution. In real peri-implantitis cases, additional factors such as saliva, blood, patient-related variables (e.g., access, compliance, soft tissue characteristics), and biofilm complexity may alter the effectiveness of mechanical decontamination. Therefore, our findings should be viewed as an indication of relative instrument performance under controlled conditions rather than absolute clinical outcomes. Clinical studies are needed to validate whether the observed efficacy translates into improved healing or disease resolution.

What has been described so far can be clearly observed in the results of the analysis of the in vitro models representing the circular bone defects at 30° and 90°, and although the data from the defects at 60° differ from these, it is still clear which two instruments perform best.

Indeed, this defect type proved to be the least operator-dependent and was, on average, the easiest to clean across all four instruments. In other words, the ultrasonic scaler, the titanium brush, the titanium curettes and erythritol air-polishing all achieved higher cleaning percentages with this type of defect compared with the other two conformations.

The explanation for this would seem to lie in Iatrou et al.’s article [20]. They argue that the 60° defect has a greater volume which allows it to be filled with water, more so than the 30° or 90° defect. As a consequence, the water is “activated” by the frequency of the instruments and reflected from the defect walls during the cleaning process, which also creates a cavitation effect with cleaning power. A further finding reported in this article supports our findings, which is that titanium curettes perform better in this type of bone defect than in others (in our study this was found to be the case with the experienced hygienist and dentist, but not with the less experienced student), which the authors explain as being possibly due to the rigidity of the mucosa, supported all around by bone walls allowing the hand to exert more regular pressure during the in vitro cleaning experiment.

Some limitations of the present study should be pointed out: firstly, the results obtained from this in vitro study should be validated with an in vivo study, which for ethical reasons would be difficult to perform in the same way. Secondly, the consistency of mucosa affected by peri-implantitis is difficult to reproduce with laboratory silicone and this may make it more difficult to manipulate the instruments, especially those requiring more space for maneuver within the sulcus. Moreover, simulating the biofilm on the implant surface with nail polish is certainly not like a real biofilm, which would be difficult to reproduce accurately in an in vitro study. Another important limitation is the potential alteration of implant surfaces following mechanical debridement. Instruments such as ultrasonic scalers, titanium brushes, and curettes may induce surface roughening, micro-scratches, or changes in surface energy, which could affect future biofilm adhesion and peri-implant tissue responses. Although the study focused on cleaning efficacy, implant surfaces were visually inspected after instrumentation, and no gross alterations (scratches or roughness) were noted; however, microscopic surface changes were not systematically evaluated. While our study focused on cleaning efficacy, future investigations should combine quantitative cleaning outcomes with surface characterization techniques (e.g., SEM or profilometry) to balance decontamination efficacy with preservation of implant surface integrity. Therefore, our results should be interpreted within the context of this model, acknowledging its limitations but also its relevance as an established experimental approach.

In clinical terms, ultrasonic scalers and titanium brushes appear to offer the most predictable decontamination performance, even for less experienced operators. However, since none of the tested approaches achieved full surface cleaning, non-surgical methods alone may be insufficient for advanced peri-implantitis. Combining mechanical debridement with adjunctive or surgical measures remains advisable to optimize clinical outcomes.

## 5. Conclusions

Within the limits of this in vitro study, none of the tested instruments achieved complete decontamination of implant surfaces. Ultrasonic scalers and titanium brushes showed higher and more consistent cleaning efficacy compared with curettes and erythritol air-polishing, and were less dependent on operator experience. Among the three defect morphologies, 60° defects appeared the most amenable to cleaning.

These results should be interpreted cautiously, as in vitro conditions cannot fully reproduce the biological and clinical complexity of peri-implantitis. Nevertheless, the findings may provide useful guidance in everyday practice, suggesting that ultrasonic scalers and titanium brushes could be considered as first-line instruments for non-surgical decontamination, while recognizing the need to combine them with adjunctive or surgical approaches in clinical settings.

## Figures and Tables

**Table 1:** Overall cleaning ability results for operators, instruments and resorption pattern.

Variable	Cleaning Percentage (Mean + SD [95% CI])
ODEXP	36.6% ± 12.5% [32.4%; 40.9%]
IGEXP	29.7% ± 11.5% [25.7%; 34.3%]
STUD	26.1% ± 10.6% [22.7%; 30.1%]
TiCu	28.14% ± 11.5% [23.3%; 32.7%]
US	35.01% ± 11% [30.7%; 39.9%]
AirPo	22.89% ± 11% [17.6%; 26.4%]
TiBr	37.16% ± 10.2% [34.7%; 43.3%]
30° defect	27.4% ± 9.3% [24.0%; 30.4%]
60° defect	37.6% ± 14% [31.5%; 41.4%]
90° defect	27.4% ± 9.9% [22.7%; 29.9%]

## Data Availability

The data supporting the findings of this study are available from the corresponding author upon reasonable request.

## Abbreviations

The following abbreviations are used in this manuscript:

STUD	Dental Hygiene Student
IGEXP	Expert Dental Hygienist (>10 years’ experience)
ODEXP	Expert Dentist (>10 years’ experience)
US	Ultrasonic Scaler
TiBr	Titanium Brush
AirPo	Air polishing with erythritol
TiCu	Titanium Curette
